# Estimates of home and leisure injuries treated in emergency departments in the adult population living in metropolitan France: a model-assisted approach

**DOI:** 10.1186/1478-7954-12-2

**Published:** 2014-02-04

**Authors:** Christophe Bonaldi, Cécile Ricard, Javier Nicolau, Maryline Bouilly, Bertrand Thélot

**Affiliations:** 1 Department of Chronic Diseases and Injuries, French Institute for Health Surveillance, 12 rue du Val d'Osne, Saint Maurice F 94 415, France; 2 North-Alps Emergency Department Network, Annecy Hospital, Annecy, France

**Keywords:** Home and leisure injuries, Hospital discharge databases, Separate ratio estimator, Poisson mixed model, Multilevel modeling, Epidemiological surveillance

## Abstract

**Background:**

Home and leisure injuries (HLIs) are currently a major public health concern, because of their frequency, associated consequences, and considerable medical costs. As in many other countries in Europe, in France the population coverage of the surveillance system of HLIs is low. In this study, a model-assisted approach is developed to estimate the incidence rates of HLIs in adults treated in emergency departments (EDs) in metropolitan France between 2004 and 2008.

**Methods:**

Using a sample of the hospitals participating in the French ED-based surveillance system, a generalized linear mixed model was applied, which describes the relationship between the numbers of ED visits for HLIs and the sex and age of the patients on the basis of the number of injury-related stays recorded by the hospitals. Statistics on hospital stays were provided by the French hospital discharge databases in the participating hospitals. The same statistics were available at the national level, which made it possible to extrapolate national incidence estimates.

**Results:**

Over the 2004–2008 period, the estimated incidence rate of HLIs age-standardized on the European population aged 15 years and over was 48.7 per 1,000 person-years (95% confidence interval: 39.4-58.0), and displayed little variability over time. This rate corresponded to an average of 2.5 million emergency hospital visits each year due to an HLI in people aged over 15 in France.

**Conclusions:**

The method made it possible to use medico-administrative datasets available nationwide to provide informative estimates despite the small number of participating EDs. The consequences and costs generated by hospital emergency visits can sometimes be onerous, and these estimated rates confirm the scale of the problem and the need to continue investing in preventive actions.

## Introduction

The term “home and leisure injuries (HLIs)” commonly refers to unintentional injuries occurring either in the home or in its immediate vicinity (garden, garage, or other outbuildings), outside (in the street, on the sidewalk), at school, or during leisure activities or sport. In other words, it is all injuries occurring at any moment during private life apart from traffic injuries, occupational injuries, suicide, violence, or assault. Home and leisure injuries correspond to a significant public health problem and are the cause of several million of deaths annually worldwide [1-3]. In France, about 20,000 deaths induced by a HLI have been identified from death certificates in 2008 [4], which was equivalent to about five times the number of deaths due to injuries sustained as a result of traffic accidents for the same year. Even in non-fatal cases, the costs to society of emergency care, hospitalizations, treatments, and the follow up of consequences are significant [5-7]. In the Netherlands, it has been estimated that for every death induced by an HLI, 38 HLIs are followed by a hospital admission, 370 require first aid treatment, and 581 lead to consulting a general practitioner [8]. For the individuals concerned, the consequences can be dramatic, with a myriad of complications and permanent disabilities in the most serious cases [9].

Although prevention campaigns are currently being carried out in all developed countries in an attempt to reduce the burden of injuries, HLIs remain a problem that both public health and research institutions neglect [1]. Unintentional injuries are generally perceived as being unpredictable, and these events are endured with resignation as unavoidable [10,11]. HLIs have a multitude of causes and circumstances, which contributes to preventing the perception of their importance and preventability. In addition, there are many different causes, each of which results in only a few injuries. Lastly, HLIs are by definition circumstantial events, which are by nature difficult to study, because the underlying circumstances are rarely known and the consequences are non-specific. All this contributes to limiting the approach to HLIs to their medical, social, and economic consequences. Some epidemiological studies have attempted to investigate the circumstances under which injuries occur, mainly either in the populations that are most at risk, i.e., children and the elderly [12-15], or focused on specific types of injury (burns, fall-induced injuries, drowning), activities (sport-related injuries), or anatomical sites. However, reliable information about the incidence of HLIs in the general population is often lacking, particularly among adults. The number of HLIs is generally either unknown at a national level or the estimates available are simply extrapolations from the raw data recorded by a small sample of emergency departments (EDs) or based on hypotheses for hospital catchment areas [16].

In France, a network of emergency rooms (“*Enquête permanente sur les accidents de la vie courante,*” EPAC), provide the surveillance coverage of HLIs. Each year, a fluctuating number of volunteer hospitals in about 10 French cities use a standardized protocol to record all HLIs treated in their EDs. The epidemiological data collected describe the circumstances and consequences of injuries and assist preventative action against HLIs in France. Until now, these data were also used to make a crude extrapolation of the incidence rates of HLIs in the French population [17] based on weak assumptions on the population coverage of the network. The aim of this paper is to propose a statistical model-based approach to predict the rates of HLIs treated in EDs in the French population, using EPAC records between 2004 and 2008 plus an auxiliary source of data available nationwide. This exogenous source is the PMSI system (“*Programme de médicalisation des systèmes d’information,*” http://www.atih.sante.fr), a medico-administrative database similar to the Medicare Diagnosis-Related Groups.

## Materials and methods

### Injury data

The EPAC surveillance network has been reporting data on HLIs since 1986. It constitutes the French component of the European Injuries Data Base (IDB, formerly designated EHLASS: European Home and Leisure Accident Surveillance System), a systematic, crossnational injury information system that provides data recorded by ED-based surveillance systems within several European Union Member States in a harmonized format [16].

When an emergency room of one of the EPAC hospitals admits a person with an injury, the medical staff collects some additional information to find out whether the injury took place at home or during a leisure activity. In particular, hospitals participating in the network receive a grant from the French Institute for Health Surveillance (“*Institut de Veille Sanitaire,*” InVS) to employ health care professionals (physicians and data input operators) to select the relevant cases and to code the data using reference guidelines [18,19]. Since 2002, each participating hospital has been implementing a quality control procedure in order to assess the reliability and exhaustiveness of the data, crosschecking the data recorded in EPAC and the information in the patient’s medical records. The personal data collection received formal approval from the French Consultative Committee for the Data Processing in Health Research (“*Comité Consultatif sur le Traitement de l'Information en matière de Recherche dans le domaine de la Santé,*” approval n°13547) and the French Commission on Individual Freedom and Data Storage (“*Commission Nationale de l’Informatique et des Libertés,*” CNIL).

**Figure 1:**
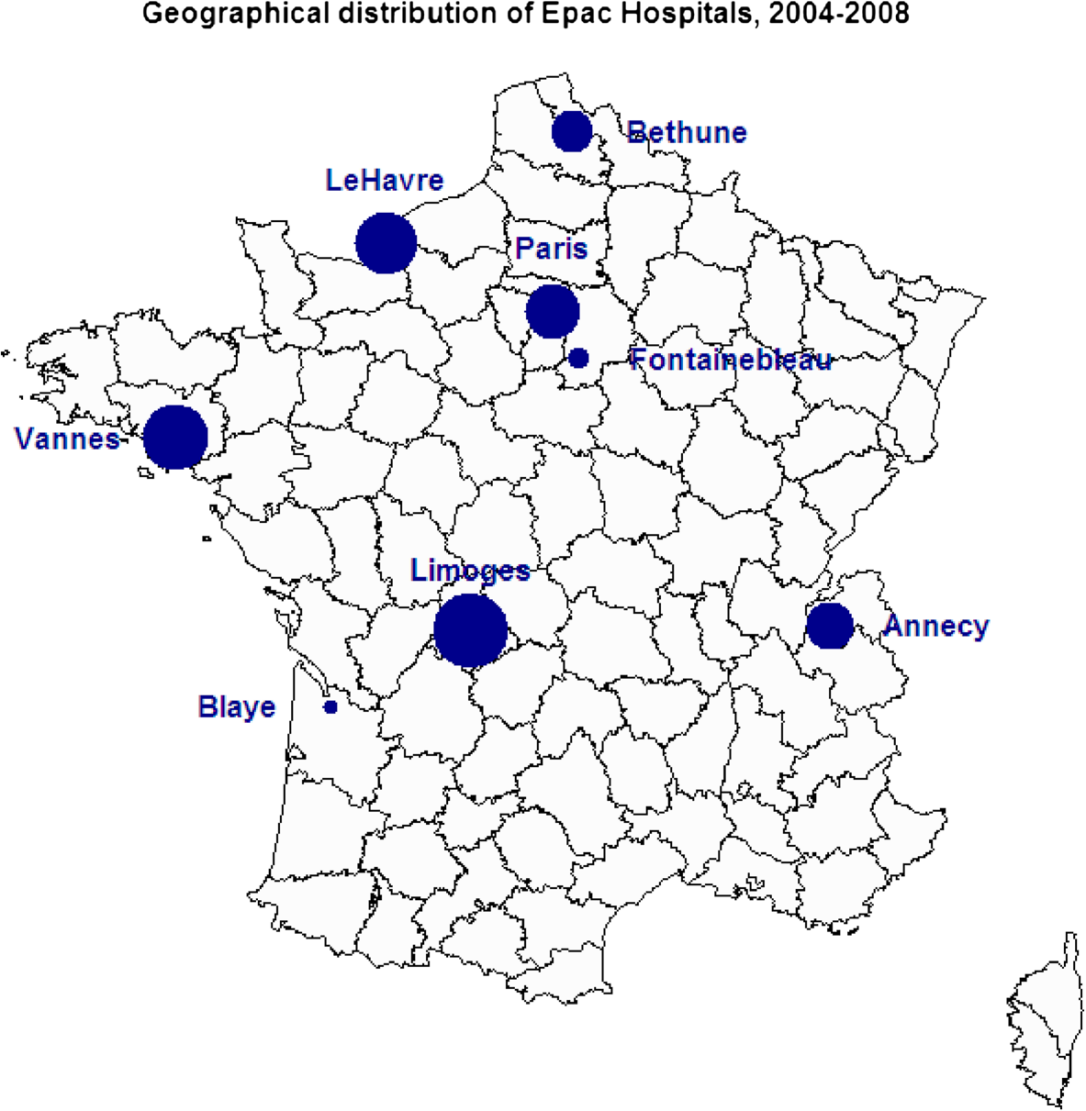
**Geographical distribution of the sample of participating hospitals from 2004 to 2008 in the EPAC network used to estimate the rate of HLIs treated in EDs in metropolitan France.** The area of the spot is proportional to the number of stays for injuries (aged over 15, reference area = Blaye).

For reasons related to the quality and homogeneity of the data, this study is based on the data collected from 2004 to 2008, the most recent years of recorded data available when the analysis was carried out. In the end, eight hospitals were included in the analysis. Full data for these five years were available for four of these hospitals (in the cities of Vannes, Bethune, Annecy, and Le Havre). In addition, data were available for Cochin Hospital (Paris) from 2005 to 2008, for Limoges Hospital from 2006 to 2008, for Blaye Hospital for 2007 and 2008, and for Fontainebleau Hospital for 2008 only (see Figure 1 for the geographical distribution of hospitals). Data were missing for some years for various reasons: the hospital was a new participant in the network, the records were incomplete, or the quality of data was not considered sufficient (generally, during the first year those records were kept). Three other hospitals in the network were excluded for the same reasons or because the recorded data concerned only pediatric EDs. Finally, the adults’ population coverage estimates of the EPAC network fluctuated from 1.5% in 2004 (with four hospitals) to 2.7% in 2008 (with eight hospitals, see Additional file 1: Table S1).

Numbers of patients over 15 years of age with HLIs admitted to a participating Emergency Department were aggregated by hospital, gender, and five-year age categories for each year.

### Auxiliary data

In France, the PMSI system records all admissions to private and public hospitals. This medico-administrative database is used primarily for managing operating costs, but it also includes medical information. Each patient stay is documented with both demographic information (age, gender, postcode of the area of residence) and clinical information (diagnosis and medical procedures). Diagnoses are coded according to the International Classification of Disease and Related Health Problems 10th Revision (ICD-10, http://www.who.int/classifications/icd). The PMSI does not record the circumstances of health events related to the hospital stay and so does not provide a useful database for studying HLIs. However, the hospital discharge database is available nationwide. We used the PMSI data to estimate the yearly volume of hospital activities related to injuries in each hospital in the EPAC network and more generally in the full set of medical establishments with an Emergency Department in metropolitan France. The InVS is allowing accessing permanently PMSI data by the CNIL (authorization n° 902167).

Injury-related hospital stays of patients aged over 15 between 2004 and 2008 were identified using two different algorithms. The first algorithm consisted of selecting all stays with a principal diagnosis code or an associate diagnosis code from Chapter 19 of ICD-10 related to “injury, poisoning and certain other consequences of external causes” (S00 - T98). The second algorithm extended the first by adding the codes of disorders strongly related to injuries (eyes: H05, H16, H26, H27, H40; ears: H72; respiratory conditions: J68-J70; skin: L55-L59).

As previously and for each algorithm, the aggregate numbers of hospital stays were determined for each year by gender and age, for each EPAC hospital, and nationally after identifying all hospitals in metropolitan France with an ED.

### Statistical modelling

The nationwide estimation of the number of HLIs treated in an ED was based on a *separate ratio estimator* of a total population size [20]. The central idea is that the number of HLIs admitted to emergency rooms in a given hospital is roughly proportional to the number of stays for injuries recorded in that hospital. In other words, the working model is:

(1) $$I_{\mathrm{ijk}}\left(t\right)=\alpha_{\mathrm{ij}.}\left(t\right)S_{\mathrm{ijk}}\left(t\right)+\epsilon_{\mathrm{ijk}}\left(t\right)$$

where *I*_*ijk*_ (*t*) is the number of HLIs recorded in the ED and *S*_*ijk*_ (*t*) the number of stays for patients of sex *i =* 1,2 in an age category *j* = 1,…,15 in hospital *k* during year *t,* where *ϵ*_*ijk*_(*t*) has a mean value of zero for the whole hospital population. The estimation of *α*_*ij.*_(*t*) at the population level relied on modeling the ratio *α*_*ijk*_(*t*) of the number of HLIs admitted to EDs to the number of injury-related stays, recorded in the sample of EPAC hospitals (*k =* 1,…,8). To take into account the hierarchical structure of the data, and the fact that the counts were repeated yearly for some hospitals, we used a generalized linear mixed model (GLMM) describing the number of HLIs with age, sex, and year of record introduced as fixed effects. Conditionally on hospital random effects, we assumed that the numbers of HLIs had a Poisson distribution, and the logarithm of numbers of stays according to age categories, sex, and year were included in the model as an offset. Age was treated as a continuous variable, taking the center of the five-year age categories. The scale of the age effect on the counts was investigated using natural cubic regression splines [21]. To investigate general patterns in the dependencies of the ratio on time (up to five equally spaced measures in increments of one year), time variable was transformed into an ordered factor with the natural orthogonal polynomial contrasts for this type of factor [22].

Standard diagnostic tools were used to assess normality assumptions on random effects and the goodness of fit of the model [21]. All analytical runs were performed using a quasi-GLMM model with the ‘MASS’ package [23] in R 2.12.0 (http://www.R-project.org), which made it possible to handle overdispersed data.

The marginal expectation of the ratio, averaged over the random effects distribution, was then inferred from the fitted model. When implementing the working model (1), the total numbers of HLIs can be provided by a combination of sex and five-year age categories. Estimates of the totals for whole population or separately by sex were computed by adding the estimated totals within the five-year age categories. In addition, the rates of HLIs treated in EDs were calculated and expressed as the number of HLIs per 1,000 inhabitants aged 15 years and over each year. Population figures for each five-year age category were available from the French National Institute for Statistics and Economics Studies datasets. Yearly rates for men, women, and both were age-standardized using the European standard population over the truncated age range of 15 years and over [24]. The variances of aggregate numbers for the whole population or by sex and the corresponding rates were estimated by the delta-method (see Appendix for more details) and used to compute 95% confidence intervals. Number estimates and age-specific rates were presented graphically (R code [25]).

The ability of the model to yield robust estimates despite the small sample size of hospitals was tested using crossvalidation reasoning. The number of HLIs and rates for the overall period 2004–2008 were estimated using the marginal expectation of the ratio obtained when one hospital was excluded. The procedure was repeated for each hospital in the network, and results were reported in the form of a *forest plot*[26,27].

## Results

### Algorithm

**Figure 2:**
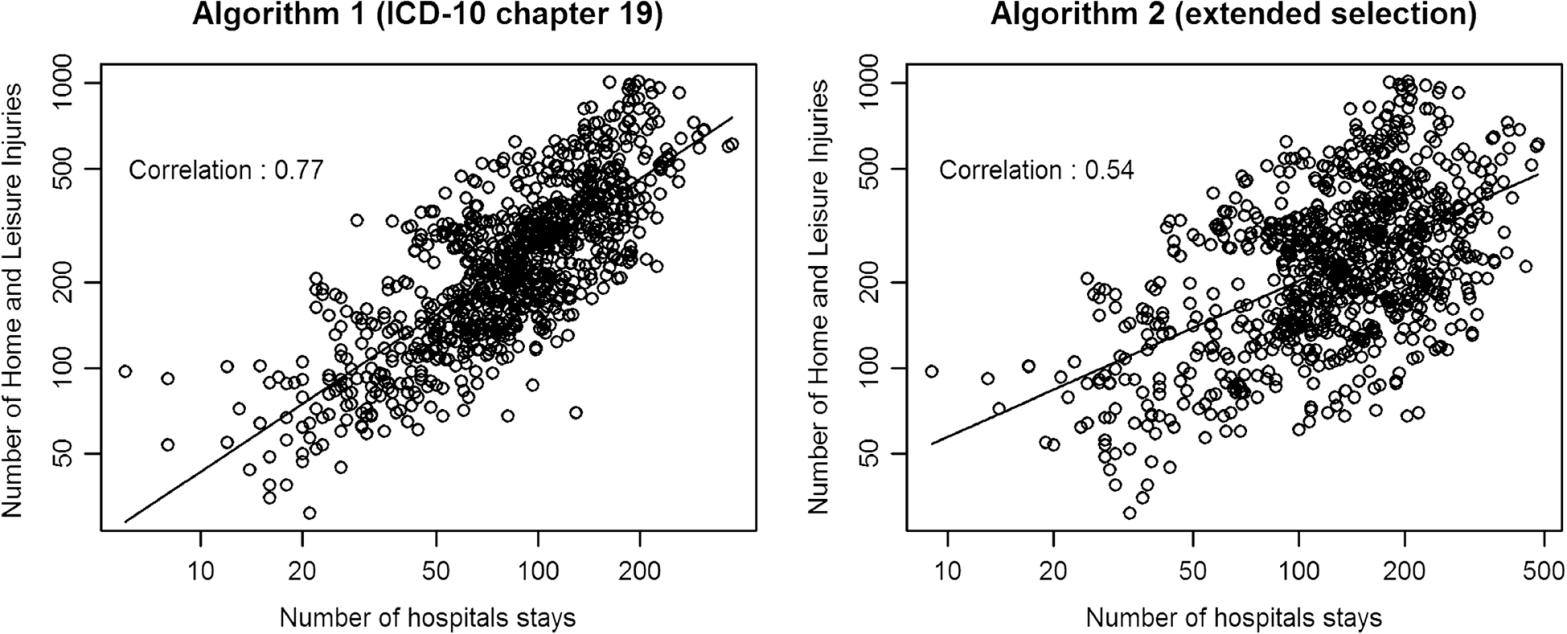
Log-scaled numbers of HLI versus log-scaled numbers of hospital stays observed in the eight hospitals of the EPAC network for each combination of sex and age category from 2004 to 2008, according to algorithm 1 (ICD-10 chapter 19 diagnostic codes) and algorithm 2 (extended selection).

In respect of the working model, we had to choose the algorithm that provided the strongest proportional relationship between the number of HLIs and the number of stays. Each point in Figure 2 shows the number of HLIs versus the number of stays for each combination of sex and age category for the eight EPAC hospitals throughout the observation period. Numbers were log-scaled to stabilize the variance according to the Poisson distribution assumption and to provide plots that are more readable. The numbers of stays, identified by the extended selection of diagnostic codes (algorithm 2, correlation coefficient = 0.54), revealed clearly higher variability than counts obtained using the restricted selection (algorithm 1, correlation coefficient = 0.77). The working model based on algorithm 1 (chapter 19 of ICD-10) gives a better approximation to the log-distribution of the data, and modeling the ratio using this selection algorithm accordingly provides estimates with lower variance. All the results reported in this paper are based on this algorithm. The yearly numbers of hospital stays identified for each EPAC hospital and overall in all hospitals in metropolitan France that have an ED are provided in the Additional file 1: Table S1.

### Final model

Using the same notations as above, the final model fitted was:

(2) $$\begin{array}{ll} \;\eta_{\mathrm{ijk}}\left(t\right)= & \mathrm{offset}\left(\log S_{\mathrm{ijk}}\left(t\right)\right)+\beta_{0}+\alpha_{i}+\delta \cdot t\\ & +\beta_{1}\cdot {Β}_{1}\left(a_{j}\right)+\beta_{2}\cdot {Β}_{2}\left(a_{j}\right)\\ & +\left(\beta_{3}+\gamma_{i}\right)\cdot {Β}_{3}\left(a_{j}\right)+b_{k}+b_{\mathrm{ijk}} \end{array}$$

$$b_{k}\sim N\left(0,{\sigma_{1}}^{2}\right)\;b_{\mathrm{ijk}}\sim N\left(0,{\sigma_{2}}^{2}\right)$$

with *η*_ijk_(t) = log(*μ*_*i*jk_(t)) and *μ*_*ijk*_(*t*) = *E*[*I*_*ijk*_(*t*) | *b*_*k*_, *b*_*ijk*_].

The intercept is represented by *β*_0_, the sex effect by *α*_*i*_, and the time slope by *δ. b*_*k*_ denotes the *hospital* random effect, and *B*_*m*_(*a*_*j*_) the age transformed in the spline basis computed at the center of the five-year age category, *j*. To handle the variability of the repeated counts reported for most hospitals, we added *b*_*ijk*_, a further random effect at level 2 of clustering (*sex-age category within hospital*), and used a meaningful first-order autoregressive *AR*(1) structure for the correlation between level 1 observations (repeated counts nested on level 2, correlation estimate at lag 1 = .38). Indeed, the auto-correlation function of the Pearson residuals from the straightforward two-level model revealed a temporal correlation pattern between repeated measurements.

Based on graphical comparison of the smoothers at the *hospital* level, we considered that a spline with three degrees of freedom (*m* = 1,…, 3) was sufficient to model the non-linear relationship with age. The knots were evenly spaced throughout the age range, with internal knots at ages 40 and 64. Only the linear trend for the time effect was retained (using time as a continuous variable), because the orthogonal polynomial contrasts were not significant. Although the time effect had a *p-*value just above the .05 level (Table 1), we kept the adjustment on the temporal trend in the final model. Interactions between sex and the age components transformed in the spline basis were introduced but only the parameter for the interaction with the third component was highly significant (*p-*value = .003) and was retained in the final model (denoted by *γ*_*i*_). This meant that the trend of the ratio versus age differed between men and women within the age range bounded by the last two knots of the spline, i.e., above age 64. Diagnostic plots used to assess the goodness of fit of the model did not suggest any serious problem (see Additional file 2: Figure S1).

**Table 1:** Estimated GLMM parameters

**Parameter**	**Estimate**	**(Std. Error)**	***p*-value**
*Fixed effects*			
*β*_*0*_ (intercept)	1.59	(.103)	<.001
*δ* (*year*)	.009	(.005)	.052
*α*_*2*_ (*woman*)	.068	(.023)	.003
*β*_*1*_ (Β_1_(*age*))	-.680	(.049)	<.001
*β*_*2*_ (Β_2_(*age*))	-1.37	(.073)	<.001
*β*_*3*_ (Β_3_(*age*))	-.394	(.060)	<.001
*γ*_*2*_ (woman*Β_3_(*age*))	-.227	(.074)	.003
*Random effects*			
*σ*_*1*_ (hospital) [IC_95%_]	.27	[.16 - .44]	
*σ*_*2*_ (unit *) [IC_95%_]	.10	[.08 - .14]	
*Auto-correlation AR*(1)			
*ρ* (lag 1) [IC_95%_]:	.38	[.26 - .49]	

*repeated measurements of counts for the same sex-age category within the hospital.

From (2), after a little calculation (see McCulloch et al. [28] and Appendix) under the normal hypothesis for random effects and given the log link, the marginal expectation of the ratio (averaged over *b*_*k*_ and *b*_*ijk*_) is given by:

(3) $$\begin{array}{ll} \;{\hat{\alpha }}_{\mathrm{ij}.}\left(t\right) & =E\left[E\left[\left(I_{\mathrm{ijk}}\left(t\right)\;\right|\;b_{k},b_{\mathrm{ijk}}\right]/S_{\mathrm{ijk}}\left(t\right)\right]\\ & =\exp\left\{\beta_{0}+\alpha_{i}+\delta \cdot t+\beta_{1}\cdot {Β}_{1}\left(a_{j}\right)\right)+\beta_{2}\cdot {Β}_{2}\left(a_{j}\right)\\ & \;\left(+\left(\beta_{3}+\gamma_{i}\right)\cdot {Β}_{3}\left(a_{j}\right)+\sigma_{1}^{2}/2+\sigma_{2}^{2}/2\right\} \end{array}$$

### Estimates

**Figure 3:**
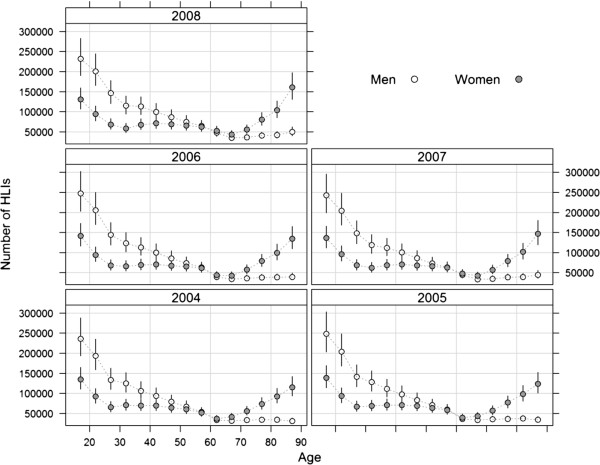
**Estimates and 95% confidence intervals of the number of HLIs treated in EDs in metropolitan France for each year from 2004 to 2008, according to age category and sex.** (Points are drawn at the center of five-year age categories).

The estimated number of HLIs in adults aged over 15 and treated in EDs of hospitals in metropolitan France rose moderately and non-significantly between the years 2004 and 2008: from 2.38 million to 2.57 million, but the numbers tended to stabilize over time (Table 2). The trend pattern was similar in both sexes. About 54% of HLIs in emergency rooms affected men each year, but the relationship between the number of HLIs and age displayed clearly different patterns in men and women (Figure 3). Among 15- to 29-year-olds, the numbers of HLIs fell sharply with age, and were significantly higher in men than in women. The number of HLIs continued to decrease steadily with age, but at a slower rate, in 30- to 64-year-old men. In the same age category, the numbers of HLIs in women showed less variability. In people older than 65 years of age, the numbers of HLIs increased strongly with age in women, whereas the numbers for men stabilized.

**Table 2:** Estimates of the numbers of HLIs (x 1,000) and age-standardized incidence rates (per 1,000 persons year) in adults aged over 15 and treated in emergency departments of hospitals in metropolitan France by year and gender

**Estimate**	**Men**	**( *IC 95% *)**	**Women**	**( *IC 95% *)**	**All**	**(*IC 95%)***
Number (*x 1,000*)						
2004	1,287	(*1,038-1,537*)	1,096	(*884*–*1,309*)	2,383	(*1,924-2,842*)
2005	1,359	(*1,097-1621*)	1,141	(*921*–*1,361*)	2,500	(*2,021-2,980*)
2006	1,382	(*1,116-1,648*)	1,161	(*938*–*1,384*)	2,543	(*2,057-3,029*)
2007	1,384	(*1,118-1,650*)	1,173	(*948*–*1,399*)	2,557	(*2,069-3,046*)
2008	1,383	(*1,117-1,650*)	1,184	(*956*–*1,413*)	2,568	(*2,077-3,060*)
Crude rates (*per 1,000*)						
2004	54.6	(*44.0-65.2*)	42.6	(*34.4-50.9*)	48.4	(*39.0-57.7*)
2005	57.2	(*46.2-68.2*)	44.0	(*35.5-52.4*)	50.3	(*40.7-59.9*)
2006	57.7	(*46.6-68.8*)	44.3	(*35.8-52.9*)	50.7	(*41.0-60.4*)
2007	57.4	(*46.4-68.4*)	44.5	(*36.0-53.1*)	50.7	(*41.0-60.4*)
2008	57.0	(*46.0-68.0*)	44.7	(*36.1-53.3*)	50.6	(*40.9-60.3*)
Standardized * rates (*per 1,000*)						
2004	55.4	(*44.6-66.1*)	39.0	(*31.4-46.5*)	47.2	(*38.1-56.3*)
2005	58.1	(*46.9*-*69.3*)	40.1	(*32.3*-*47.8*)	49.1	(*39.7*-*58.5*)
2006	58.6	(*47.3*-*69.8*)	40.2	(*32.4*-*47.9*)	49.4	(*39.9*-*58.8*)
2007	58.3	(*47.1*-*69.5*)	40.0	(*32.3*-*47.7*)	49.1	(*39.7*-*58.5*)
2008	57.9	(*46.7*-*69.0*)	39.7	(*32.1*-*47.4*)	48.8	(*39.5*-*58.2*)

*Age-standardized using the European standard population over the truncated age range of 15 years and over.

**Figure 4:**
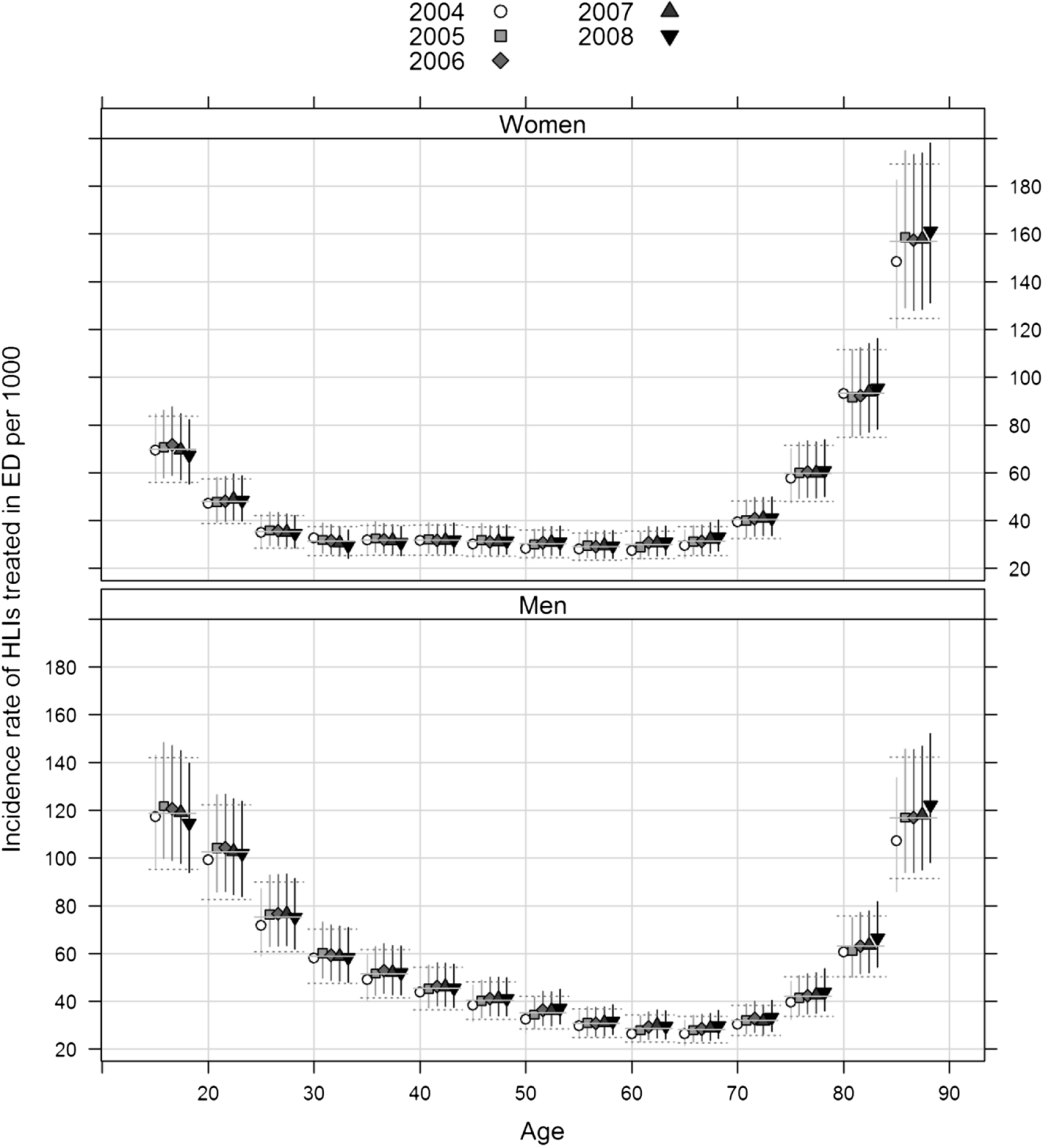
**Estimates and 95% confidence intervals of metropolitan age-specific incidence rates of HLIs treated in emergency departments from 2004 to 2008, for women and men.** Horizontal segments represent age-specific rates for the period 2004–2008 (solid line: estimates, dotted line: 95% confidence intervals).

Overall, rate estimates of HLIs stayed relatively stable over time (Table 2). Over the 2004–2008 period, the estimated age-standardized rate of HLIs on the truncated age range (15 years and over) European population was 48.7 (IC_95%_ = [39.4-58.0]). The age-standardized rate in men (57.6, IC_95%_ = [46.6-68.7]) was higher than in women (39.8, IC_95%_ = [32.1-47.4]), with a significant standardized rate ratio of 1.45 (IC_95%_ = [1.09-1.92]). Age–specific incidence rates both for each year and over the period 2004–2008 are reported in Figure 4. In men, rates were maximal at younger and older age categories. In women, the incidence peak was reached in older women, with a very high rate in 85 years and over (157 in 2004–8, IC_95%_ = [125–189])

**Figure 5:**
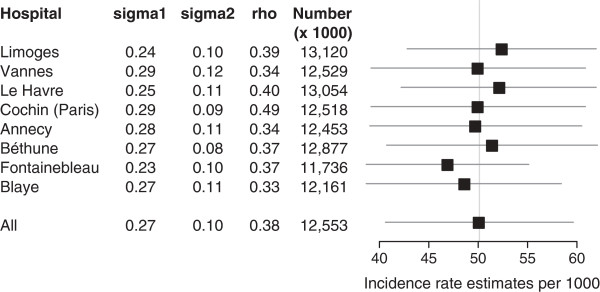
**Forest plots of estimates of random effects (sigma1 =** ***σ***_**1 **_**(hospital)), sigma2 =** ***σ***_**2 **_**(sex-age category within hospital level), auto-correlation (rho =** ***ρ *(lag1)), total numbers of HLIs over the 2004–2008 period, and related rates with 95% confidence intervals, when excluding the hospital named in the first column.** The vertical line indicates the central estimates for the full sample of hospitals (last row).

Figure 5 shows the results of the crossvalidation process comparing the estimates based on the full analysis set of hospitals to those obtained when one hospital was excluded from the sample. The relative difference from the full sample rate estimate ranged from -6% (exclusion of Fontainebleau hospital) to +5% (exclusion of Limoges hospital). All estimates were within the confidence interval of the full sample rate estimate.

## Discussion

The goal of this study was to find a way to estimate the numbers of HLIs in adults treated in ED hospitals throughout metropolitan France. This national number of HLIs is difficult to estimate because in France, as in most European countries, the HLI surveillance system is based on data from the EDs of a restricted number of hospitals. To achieve our goal, we used a model-assisted approach that combined data available from the ED surveillance system with information available at the population level (hospital discharge statistics).

Based on this approach, we estimated a yearly average number of HLIs in the population over 15 years of age of 2.5 million between 2004 and 2008. This number of HLIs in adults accounted for 15% of ED activity in metropolitan France (without any age limit), with about 17 million ED visits recorded each year [29]. The estimated numbers of HLIs showed a slight increase over time (+190,000 between 2004 and 2008), but age-adjusted rates of HLIs treated in emergency rooms varied little over time. The pattern of the numbers of HLIs versus age was markedly different in men and women, which was consistent with the knowledge.

There are few recent reports of the number of HLIs in France, and comparisons with earlier French published results are flawed due to the use of variable methods, especially concerning the data collection setting, targeted population, and even the definition of HLIs [17,30-32]. In Europe, crosscountry comparisons of ED visit rates due to HLIs suggest that the rate in France could be amongst the lowest, with a level roughly comparable to that in the Netherlands [33]**]**. The widespread access to primary care with a low cost burden for the patient in France may contribute to the treatment of minor injuries by primary care providers, such as general practitioners, and therefore to reduce the uptake of ED treatment. However, the interpretation of rate differences between European countries has been shown to be somewhat hazardous [33]. In practice, data are collected without any formal sampling scheme, and are markedly influenced by the characteristics of the national health care system. In addition, extrapolated rates from coverage estimates are provided without confidence intervals in most countries, which does not help in interpreting discrepancies. Nevertheless, some common tendencies do appear, such as the similar age pattern of HLI rates in Denmark, Netherlands, Norway, England, and Wales [33,34] and these were consistent with our findings.

To some extent, both minor injuries and very serious injuries (directly rushed into hospital), escape detection by ED-based surveillance. In addition, despite quality control processes in most ED-based surveillance systems [33], some cases may be missed, for instance if the circumstance of the event are ambiguous and this leads to the misclassification of the injury. Consequently, rate estimates definitely provide an underestimated indicator of the real burden of HLIs. However, in the EPAC network, completeness estimates are particularly high (over 95%) for our hospital sample, and the bias induced in our rate estimates by this minor lack of completeness is probably insignificant relative to their accuracy.

Our estimates show that the number of HLIs rose slightly during the five years covered by the study, mainly in the age categories from 65 years. However, after age adjustment, the rates of HLIs no longer display a clear time trend. We suggest that this can be explained by the aging of the population alongside the increased risk of HLIs in older people. In Finland, a trend analysis of fall-induced injuries, the most common cause of HLIs in older people, showed the dramatic increase in rates between 1970 and 1995 in the 50 years and older population [15]. However, if a clearly increasing trend can be identified over a long period of time (25 years in the previous study), our finding of only a slight increase is not unexpected from records covering just five years.

### Limits and strength

Our study provides the first estimates of the numbers and incidence rates of HLIs occurring in recent years at the national level in adults living in metropolitan France. However, our estimates were based on data from a limited number of emergency rooms, a common shortcoming of ED-based surveillance systems. Although the participating hospitals were roughly equally distributed geographically over the mainland territory of metropolitan France (Figure 1), they cover a variety of situations: one is located in the greater Paris area, one in a mountainous country area, four in medium-sized towns, and two in semirural areas. In addition, the analysis of the hospital distribution using yearly admission statistics shows than metropolitan hospitals in the same range as the EPAC participating sample account for 70% of the total number of hospital stays recorded in France (data not shown, see Additional file 3: Figure S2). Consequently, although we have no means to conclude absolutely on the representativeness, we do not think there is any reason to suspect a major lack of representativeness in our data regarding the ratio of HLIs to hospital stays. Moreover, results of the crossvalidation procedure show that these estimates are relatively robust regardless of the subsample used.

Based on the assumed proportion of coverage of the ED system, it would seem to be easy to extrapolate numbers or rates to the national level, as done by some European contributors to the IDB [33,35]. However, confidence intervals cannot be computed rigorously in this way, and intervals from variance estimates based on a simple assumption of random sampling are clearly strongly underestimated [17,36]. However, providing confidence intervals to assist in the interpretation of estimate reliability is all the more critical, because the number of participating centers in ED-based surveillance systems is often small. The methodological approach suggested in this present study is based on an idea similar to the extrapolated estimate: using a key hospital statistic available at the national level and constructing an age- and sex-specific “multiplier”. However, our aim was not to estimate a coverage proportion, but rather the ratio between the number of HLIs treated in EDs and the number of stays in the same hospital. Hierarchical modeling was carried out to model this ratio in order to provide statistical inference at the population level and to correctly estimate the between-hospital variability. This enabled us to provide incidence rates with confidence intervals, with at the end a somewhat suboptimal relative precision of ± 20%. However, given the sample size of the French ED surveillance system, this accuracy at the national level is what would be expected.

Our study focused solely on adults, even though children under 15 years of age account for a substantial fraction of HLI victims. Including children would not have made it possible to provide reliable national estimates for children from the EPAC data. In France, several emergency systems coexist to deal with pediatric emergencies: non-specialized EDs, non-specialized EDs linked to a pediatric structure, and fully pediatric-specific EDs [29]. Between 2004 and 2008, the EPAC surveillance network collected data from only two pediatric EDs (Hospitals in the cities of Besançon and Marseille). Moreover, the children sent to non-specialized EDs may be referred to a nearby pediatric ED. In addition, whether a child is hospitalized or not probably depends very considerably on whether a specific pediatric structure is available. Consequently, the ratio between the number of HLIs and the number of stays in children displays strong overdispersion, which makes it impossible to provide robust estimates using the statistical approach described here. Consistently, the ED-based rates of HLIs in children in Europe display particularly high variability [33], perhaps for these very reasons. Determining the nationwide incidence of ED visits due to HLIs in children is therefore still a challenge.

## Conclusion

It is still difficult to assess the burden resulting from HLIs at the population level in Europe, because of the limited size of the samples provided by ED surveillance systems. The model-based approach provided in this paper makes it possible to compute rate estimates of HLIs treated in EDs at the national level, together with confidence intervals consistent with the small number of participating EDs. The method is simple to implement, and is probably applicable to other national ED surveillance systems with similarly low coverage rates. Given the sometimes onerous consequences and costs generated by hospital emergency visits, the estimates of the rates of HLIs treated in EDs provided by this study highlight the extent of the problem and the need to continue to invest in preventive actions.

## Appendix

### Delta method estimates for the aggregated numbers

Without loss of generality, the computing for the total number of cases is provided. But it can be straightforwardly reproduced on subgroups (by sex for example) or generalized to approximate the variance of incidence rates, which are just a weighting sum of the numbers.From equation (3), the total numbers of HLIs by combination of sex and five-year age categories are

$${\hat{I}}_{\mathrm{ij}.}\left(t\right)={\hat{\alpha }}_{\mathrm{ij}.}\left(t\right)\cdot S_{\mathrm{ij}.}\left(t\right).$$

It follows the whole number

$${\hat{N}}_{\mathrm{ij}.}\left(t\right)=\sum_{\begin{array}{l} i\\ j \end{array}}{\hat{\alpha }}_{\mathrm{ij}.}\left(t\right)\cdot S_{\mathrm{ij}.}\left(t\right)$$

or in vector formulation

$$\begin{array}{ll} \;{\hat{N}}_{\mathrm{ij}.}\left(t\right) & =\sum_{\begin{array}{l} i\\ j \end{array}}\exp\left(X_{\mathrm{ij}}\left(t\right)\cdot \beta \right)\cdot S_{\mathrm{ij}.}\left(t\right)\\ & =g\left(\beta \right) \end{array}$$

where $\beta =\left(\beta_{0},\alpha_{i},\delta ,\beta_{1},\beta_{2},\beta_{3},\sigma_{1}^{2},\sigma_{2}^{2}\right)$ is the vector of parameters and *X*_*ij*_(*t*) = (*x*_*ij*_(*t*), 1/2, 1/2) with *x*_*ij*_(*t*) the vector of regressors.Because the total number of cases is a nonlinear function of *β*, we used the delta-method equation to approximate the variance of ${\hat{N}}_{\mathrm{ij}.}\left(t\right)$:

$$V\left({\hat{N}}_{\mathrm{ij}.}\left(t\right)\right)≅{\left(\frac{\partial g}{\partial x}\right)}_{\left|\beta \right)}^{'}V\left(\beta \right){\left(\frac{\partial g}{\partial x}\right)}_{\left|\beta \right)}$$

where *V*(*β*) is the variance-covariance matrix of parameters and ${\left(\frac{\partial g}{\partial x}\right)}_{\left|\beta \right)}$ the column-vector of partial derivatives of *g*(⋅) evaluated at *β*. For the component *m*, we have:

$$\begin{array}{ll} \;{\frac{\partial g}{\partial \beta^{\left(m\right)}}}_{\left|\beta \right)} & =\sum_{\begin{array}{l} i\\ j \end{array}}X_{\mathrm{ij}}^{\left(m\right)}\left(t\right)\exp\left(X_{\mathrm{ij}}\left(t\right)\cdot \beta \right)\cdot S_{\mathrm{ij}.}\left(t\right)\\ & =\sum_{\begin{array}{l} i\\ j \end{array}}X_{\mathrm{ij}}^{\left(m\right)}\left(t\right){\hat{I}}_{\mathrm{ij}.}\left(t\right) \end{array}$$

### Marginal effects for a Poisson GLMM

Using conventional notations in the more general framework of GLMM (see McCulloch, Searle and Neuhaus [28]), the Poisson mixed model can be written:

$$y_{i}\left|u∼Ρ\left(\mu_{i}\left|u\right)\right)\right),\;\mu_{i}=E\left(y_{i}\left|u\right)\right)$$

$$g\left(\mu_{i}\right)=x_{i}^{\prime }\beta +z_{i}^{\prime }u$$

with *g*(⋅) = log(⋅) the link function, $x_{i}^{'}$ the *i*^th^ row of the model matrix for the fixed effects, *β* the fixed effects parameters vector, *u* the random effects vector, and $z_{i}^{\prime }$ the *i*^th^ row of the model matrix for the random effects.Given the log link, we have *g*^- 1^(⋅) = exp(⋅) and:

$$\begin{array}{ll} \;E\left(y_{i}\right) & =E\left[E\left[y_{i}\left|u\right)\right]\right]\\ & =E\left[\exp\left(x_{i}^{\prime }\beta +z_{i}^{\prime }u\right)\right]\\ & =\exp\left(x_{i}^{\prime }\beta \right)M_{u}\left(z_{i}\right) \end{array}$$

where *M*_*u*_(*z*_*i*_) is the moment generating function of *u* evaluated at *z*_*i*_. In addition, if we assign a multivariate normal distribution to the random effects with mean 0 and variance covariance matrix D, we have:

$$E\left(y_{i}\right)=\exp\left(x_{i}^{\prime }\beta \right)\exp\left(z_{i}^{\prime }D\;z_{i}/2\right)$$

Equation (3) is the result of this equality.

## Abbreviations

CNIL: *Commission Nationale de l’Informatique et des Libertés*; ED: Emergency department; EHLASS: European Home and Leisure Accident Surveillance System; EPAC: *Enquête Permanente sur les Accidents de la vie Courante*; HLIs: Home and leisure injuries; ICD-10: International Classification of Disease and Related Health Problems 10th Revision; IDB: European injuries database; InVS: *Institut de Veille Sanitaire*; GLMM: Generalized linear mixed model; PMSI: *Programme de Médicalisation des Systèmes d’Information.*

## Competing interests

The authors declare that they have no competing interests.

## Authors' contributions

CB conceived of the study, performed the statistical analysis, interpreted the data, and drafted the manuscript. CR contributed to management of data collection from EPAC, provided administrative and material support, and revised the manuscript. JN contributed to management of the PMSI database, interpretation of results, and revision of the manuscript. MB participated in the data collection and provided material support. BT designed EPAC, managed data acquisition, interpreted data, and helped to draft the manuscript. All authors read and approved the final version of the manuscript.

## Supplementary Material

Additional file 1: Table S1 ED-recorded HLIs and hospital stays in EPAC participating hospitals and in mainland. Click here for file

Additional file 2: Figure S1 Model checking plots for the model (2) of the ratio. (*a*) Relationship between raw data and fitted values of the ratio. (*b*) Pearson residuals against fitted values with a Lowess curve (dashed line). (*c*) *QQ* plots for the predicted random effects at hospital level. (*d*) *QQ* plots for the predicted random effects at level 2 (*sex-age category within hospital*). (*e*) *QQ* plots for the Pearson residuals. (*f*) Boxplot of the residuals for each hospital with the random intercept estimates. These diagnostic plots suggest that model (2) is reasonable. A few observations seem to be outliers but do not influence the fit. Click here for file

Additional file 3: Figure S2 Distribution of metropolitan hospitals with an ED according to stay numbers for injuries (patients aged over 15) in 2008. The cumulated number of stays from hospitals within the range of stays recorded by EPAC hospitals (tick marks on *x*-axis) corresponded to 70% of all stays recorded in metropolitan France within the year. Click here for file
