# Supplementary material for: Estimates of home and leisure injuries treated in emergency departments in the adult population living in metropolitan France: a model-assisted approach
Source: Popul Health Metr. 2014 Feb 4;12:2. doi: 10.1186/1478-7954-12-2 (PMC3923095; doi:10.1186/1478-7954-12-2)
Supplement: Additional file 1: Table S1 — ED-recorded HLIs and hospital stays in EPAC participating hospitals and in mainland. [file 1478-7954-12-2-S1.docx]

Supplementary Data

| Table S1: ED-recorded HLIs and hospital stays* in EPAC participating hospitals and in mainland France, from 2004 to 2008 | | | | | | |
| --- | --- | --- | --- | --- | --- | --- |
| Participating hospitals | Recorded cases | Years | | | | |
|  |  | 2004 | 2005 | 2006 | 2007 | 2008 |
| Vannes | HLIs | 11,184 | 11,345 | 11,811 | 11,539 | 11,938 |
|  | Stays | 3,648 | 3,416 | 3,542 | 3,670 | 3,727 |
| Annecy | HLIs | 9,964 | 9,530 | 10,388 | 10,608 | 11,363 |
|  | Stays | 2,817 | 2,959 | 3,081 | 2,857 | 2,733 |
| Le Havre | HLIs | 7,247 | 7, 722 | 7,754 | 7,508 | 7,773 |
|  | Stays | 2,969 | 3,140 | 3,595 | 3,708 | 3,458 |
| Béthune | HLIs | 4,909 | 5,429 | 5,727 | 5,793 | 5,618 |
|  | Stays | 1,925 | 2,015 | 2,189 | 2,257 | 2,318 |
| Paris (Cochin) | HLIs | _ | 9,735 | 9,533 | 9,112 | 9,296 |
|  | Stays |  | 3,257 | 3,141 | 3,054 | 3,161 |
| Limoges | HLIs | _ | _ | 8,132 | 8,467 | 8,618 |
|  | Stays |  |  | 3,790 | 3,940 | 4,291 |
| Blaye | HLIs | _ | _ | _ | 3,130 | 2,996 |
|  | Stays |  |  |  | 798 | 761 |
| Fontainebleau | HLIs | _ | _ | _ | _ | 5,283 |
|  | Stays | _ | _ | _ | _ | 1,141 |
|  |  |  |  |  |  |  |
| *Mainland France* | *Stays* | *755,726* | *788,226* | *798,116* | *799,663* | *802,970* |
| *Population coverage estimate of EPAC network* † | | *1.5%* | *1.9%* | *2.4%* | *2.5%* | *2.7%* |
| * Stays selected on the basis of a principal diagnosis or an associate diagnosis code from chapter 19 of the ICD-10 related to “injury, poisoning and certain other consequences of external causes” (S00 - T98).  † Assessed as the numbers of stays for injuries recorded in the network divided by the corresponding statistic on the national level. | | | | | | |
