# Supplementary material for: Estimates of home and leisure injuries treated in emergency departments in the adult population living in metropolitan France: a model-assisted approach
Source: Popul Health Metr. 2014 Feb 4;12:2. doi: 10.1186/1478-7954-12-2 (PMC3923095; doi:10.1186/1478-7954-12-2)
Supplement: Additional file 2: Figure S1 — Model checking plots for the model (2) of the ratio. (a) Relationship between raw data and fitted values of the ratio. (b) Pearson residuals against fitted values with a Lowess curve (dashed line). (c) QQ plots for the predicted random effects at hospital level. (d) QQ plots for the predicted random effects at level 2 (sex-age category within hospital). (e) QQ plots for the Pearson residuals. (f) Boxplot of the residuals for each hospital with the random intercept estimates. These diagnostic plots suggest that model (2) is reasonable. A few observations seem to be outliers but do not influence the fit. [file 1478-7954-12-2-S2.docx]

**Supplementary Data**

Figure S1: Model checking plots for the model (2) of the ratio. (*a*) Relationship between raw data and fitted values of the ratio. (*b*) Pearson residuals against fitted values with a Lowess curve (dashed line). (*c*) *QQ* plots for the predicted random effects at hospital level. (*d*) *QQ* plots for the predicted random effects at level 2 (*sex-age category within hospital*). (*e*) *QQ* plots for the Pearson residuals. (*f*) Boxplot of the residuals for each hospital with the random intercept estimates. These diagnostic plots suggest that model (2) is reasonable. A few observations seem to be outliers but do not influence the fit.
