# Supplementary material for: Estimates of home and leisure injuries treated in emergency departments in the adult population living in metropolitan France: a model-assisted approach
Source: Popul Health Metr. 2014 Feb 4;12:2. doi: 10.1186/1478-7954-12-2 (PMC3923095; doi:10.1186/1478-7954-12-2)
Supplement: Additional file 3: Figure S2 — Distribution of metropolitan hospitals with an ED according to stay numbers for injuries (patients aged over 15) in 2008. The cumulated number of stays from hospitals within the range of stays recorded by EPAC hospitals (tick marks on x-axis) corresponded to 70% of all stays recorded in metropolitan France within the year. [file 1478-7954-12-2-S3.docx]

**Supplementary Data**
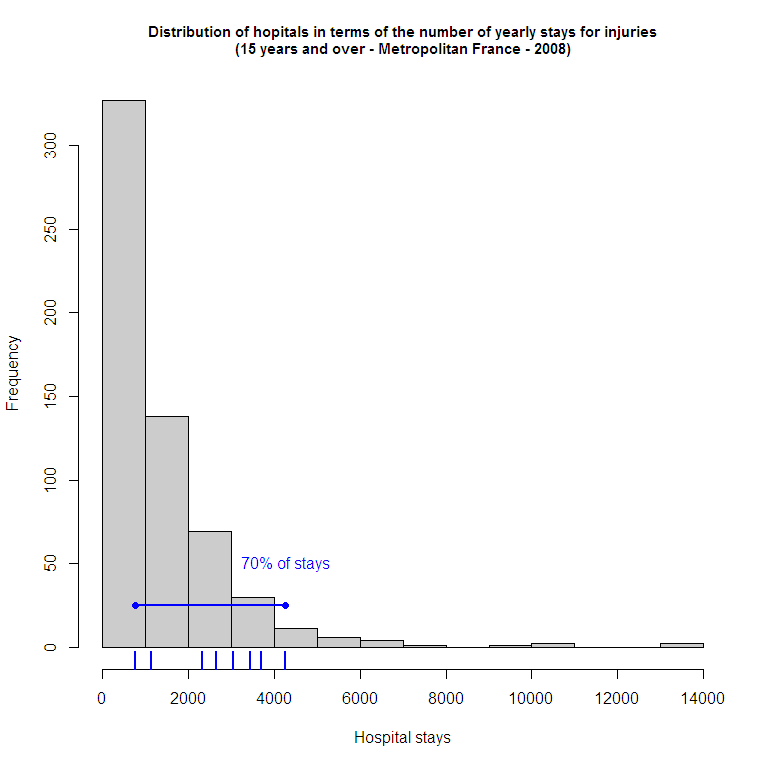


Figure S2: Distribution of metropolitan hospitals with an ED according to stay numbers for injuries (patients aged over 15) in 2008. The cumulated number of stays from hospitals within the range of stays recorded by EPAC hospitals (tick marks on *x*-axis) corresponded to 70% of all stays recorded in metropolitan France within the year.
